# Heterozygous expression of Cre recombinase in podocytes has no impact on the anti‐glomerular basement membrane glomerulonephritis model in C57BL/6J mice

**DOI:** 10.14814/phy2.15443

**Published:** 2022-09-09

**Authors:** Cyril Mousseaux, Tiffany Migeon, Perrine Frère, Marie Christine Verpont, Elisabeth Lutete, Claire Navarro, Liliane Louedec, Juliette Hadchouel

**Affiliations:** ^1^ Institut National de la Santé et de la Recherche Médicale (INSERM) Unité Mixte de Recherche Scientifique 1155, Tenon Hospital Paris France; ^2^ Faculty of Medicine Sorbonne University Paris France

**Keywords:** crescentic glomerulonephritis, mouse models, *NPHS2‐Cre*, NTS

## Abstract

A recent article described a thickening of the glomerular basement membrane (GBM) along with changes in the expression of key components of the extracellular matrix in 6‐month‐old *NPHS2‐Cre* transgenic mice, which express the Cre recombinase specifically in podocytes. This transgenic line has been widely used to characterize the implication of candidate genes in glomerular diseases in younger mice. Using a different mouse strain (C57BL/6J) than the previous report (129S6/SvEvTac), we sought to characterize 3‐ and 6‐month‐old *NPHS2‐Cre* ^*+/−*^ mice in control and pathological conditions. At baseline, there was no difference in renal function and histology between control and *NPHS2‐Cre* ^*+/−*^ mice. Notably, GBM thickness evaluated by transmission electron microscopy was similar between the two groups. We then induced an immune‐mediated severe glomerular insult, the anti‐glomerular basement membrane glomerulonephritis model (anti‐GBM‐GN) in 3‐month‐old control and *NPHS2‐Cre* ^*+/−*^ mice. *NPHS2‐Cre* ^*+/−*^ mice exhibited the same alterations in renal function and structure as control mice. In summary, our study strongly suggests that *NPHS2‐Cre* ^*+/−*^ transgenic mice on a C57BL/6J background can be safely used for podocyte‐specific gene inactivation in control conditions and in the anti‐GBM‐GN model.

## Graphical abstract

A 2019 article reported the cellular and molecular modifications induced by expression of the Cre recombinase in podocytes of 129Sv mice. We show here that the same transgene on a C57BL/6J backroung does not alter podocyte biology in either physiological or pathological conditions.

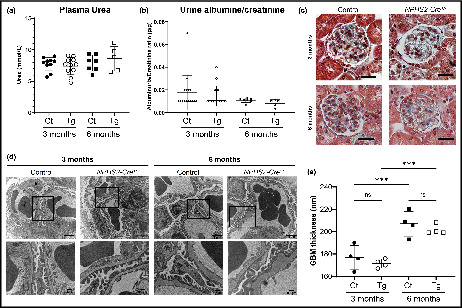

## 1 INTRODUCTION

Since its discovery in 1981, the Cre recombinase system has grandly facilitated genome editing (Sternberg & Hamilton, 1981). Adapted from P1 bacteriophages, this enzyme recognizes short palindromic DNA sequences called LoxP and then excises DNA fragments between two LoxP sites inserted in the same direction (Sauer & Henderson, 1988). Therefore, it is widely used to perform conditional (cell‐specific or inducible) gene inactivation. Tissue‐specific gene inactivation can be achieved upon two requirements: (1) The Cre recombinase must be expressed under the dependence of a cell‐specific promoter and (2) exons of the gene of interest must be flanked by two LoxP sequences.

For a long time, the question of Cre recombinase side‐effects has not been considered, to such an extent that many investigators did not include mice expressing Cre without the floxed alleles in the control group. However, a growing number of studies report that Cre recombinase can alter the phenotype of mice (Balkawade et al., 2019; Jeannotte et al., 2011; Lee et al., 2006; Loonstra et al., 2001; Schmidt et al., 2000). Among these studies, a recent paper by Balkawade et al. (2019) highlighted for the first time a dose‐dependent toxicity of the Cre recombinase in control *NPHS2‐Cre* mice. This mouse line, originally developed by Moeller et al. (2003), is widely used to study the role of a given gene in podocytes. The *NPHS2‐Cre* transgene comprises the Cre coding sequence under the control of 2.5 kb of the human *NPHS2* promoter. The *NPHS2* gene encodes Podocin, which is specifically expressed in podocytes. In their paper, Balkawade et al. observed a thickening of the glomerular basement membrane (GBM) in 24‐week‐old *NPHS2‐Cre* mice, which was not associated with any modification of glomerular/renal function. They also found that the expression of genes involved in extracellular matrix production and cell survival was deregulated in *NPHS2‐Cre* glomeruli. This work was followed by another publication in which induction of Cre recombinase in podocytes by doxycycline administration a few days after birth resulted in focal segmental glomerulosclerosis (Frahsek et al., 2019). Importantly, these two works have used the *NPHS2‐Cre* mouse line on a 129S6/SvEvTac genetic background.

Since the *NPHS2‐Cre* mouse line is routinely used in our laboratory for conditional gene targeting in podocytes, we sought to verify whether or not our mice showed the same phenotype as described above. In contrast to the study published by Balkawade et al., we generally use 2‐ to 4‐month‐old heterozygous animals on the C57BL/6J genetic background. Because the Cre recombinase toxicity is described as both dose‐ and time‐dependent, we hypothesized that the probability to observe a phenotype should be lower in our experiment. We also wondered whether or not the induction of a glomerulopathy might unmask a more severe disease in *NPHS2‐Cre* ^*+/−*^ mice despite a similar phenotype at baseline. In this study, we examined the potential toxicity of Cre recombinase in a model of rapidly progressive glomerulonephritis (RPGN), called anti‐glomerular basement membrane glomerulonephritis (anti‐GBM‐GN) (Salant & Cybulsky, 1988). RPGN is characterized by podocyte injury, activation of parietal epithelial cells leading to crescent formation, and infiltration of inflammatory cells within the kidney. This leads to acute kidney injury associated with active urinary sediment (proteinuria and hematuria). Most RPGN etiologies are antibody‐mediated as in Goodpasture disease (McAdoo & Pusey, 2017), ANCA‐associated vasculitis (Jennette & Nachman, 2017), IgA nephropathy, or lupus nephritis (Almaani et al., 2017). The hypothesis of a podocyte‐parietal epithelial cell cross‐talk in RPGN being increasingly reported (Dai et al., 2013; Pace et al., 2021), it seemed important to verify that *NPHS2‐Cre* ^*+/−*^ mice did not exacerbate the glomerular damage of anti‐GBM‐GN.

We thus found that *NPHS2‐Cre* ^*+/−*^ mice had no alteration in their function, pathology, and ultrastructure at baseline at 3–4 months of age. We also found that heterozygous expression of podocyte Cre recombinase does not impact the severity of the anti‐GBM‐GN model. In conclusion, our study suggests that *NPHS2‐Cre* ^*+/−*^ mice on a C57BL/6J genetic background represent a valid transgenic model to characterize the consequences of gene inactivation in podocytes in these conditions.

## 2 MATERIALS AND METHODS

### 2.1 *NPHS2‐Cre* ^*+/−*^ mice

All studies were performed in accordance with the European Communities Council Directive. The project has been approved by the French Ministry of Research (#22643). Animals were maintained under routine vivarium conditions, in a pathogen‐free animal facility, in ventilated cages with a 12/12 photoperiod, at 22 ± 2°C with a 55 ± 10% humidity.

*NPHS2‐Cre* mice were purchased from the Jackson Laboratory (JAX stock #008205). The genotype was determined by PCR using the following primers: Cre‐S (5′‐CCAGCTCAACATGCTGCACA‐3′) and Cre‐AS (5′‐GCCACACCAGACACAGAGAT‐3′). For all experiments, we used heterozygous *NPHS2‐Cre* ^*+/−*^ mice and their non‐transgenic littermates as controls.

### 2.2 Nephrotoxic serum nephritis model

Passive anti‐glomerular basement membrane glomerulonephritis (anti‐GBM‐GN) was induced using decomplemented sheep anti‐rat GBM serum prepared as previously described in 2‐ to 4‐months‐old male and female mice (Mesnard et al., 2009). The animals received 3 retro‐orbital intravenous injections of decomplemented nephrotoxic serum (NTS) for 3 consecutive days. The first injection consisted of a pre‐immunization dose of 50 μl of NTS mixed with 50 μl of NaCl 0.9%. The second and third injections consisted of 9 μl/g of NTS.

Urine was collected before and 10 days after the first injection. On Day 10 after the first injection, 300 μl of blood was collected by retro‐orbital puncture under general anesthesia (mixture of ketamine (100 mg/kg) and xylazine (10 mg/kg) diluted in sterile saline to a final volume of 100 μl/10 g body weight, administered intraperitoneally). Mice were then euthanized by cervical dislocation. The kidneys were collected and cut into three segments, which were either snap‐frozen in liquid nitrogen for RNA and protein extraction, or fixed in AFA (Alcohol‐Formalin‐Acetic Acid) overnight before paraffin embedding for histological and immunohistological analysis.

### 2.3 Assessment of renal function and albuminuria

Plasma urea concentration was measured using a Konelab enzymatic‐spectrophotometric analyzer (Thermo Fisher Scientific, Waltham, MA). Albuminuria was determined with an AU 480 Chemistry Analyzer (Beckman Coulter, Fullerton, California). Creatininuria was assessed via a colorimetric assay based on Jaffe's reaction with an AU spectrophotometer (SAFAS, Monaco).

### 2.4 Histopathology and immunohistochemical studies

Kidneys were immersed in AFA (Acetic acid–Formalin–Alcohol) and embedded in paraffin. Sections (3‐μm thick) were processed for histopathology study or immunohistochemistry. For histopathology, sections were stained with Masson trichrome. All glomeruli of the sections were analyzed. For each mouse, the proportion of crescentic glomeruli (defined as a glomerulus exhibiting more than one layer of cells in Bowman space) was calculated.

Immunohistochemical studies were performed on AFA‐fixed paraffin‐embedded sections. After being deparaffinized, sections were incubated for 20 min in the target retrieval solution citrate pH 6 (S2369, Dako, Agilent Technologies, Santa Clara, CA) at 100°C in a pressure cooker (TintoRetriever, BioSB, Santa Barbara, CA). They were then incubated in peroxidase blocking reagent (S2023, Dako, Agilent Technologies, Santa Clara, CA) for 10 min, blocked in TBS‐0.1% Tween (TBST) containing 10% bovine serum albumin (BSA, Euromedex, Souffelweyersheim, France), and immunostained against CD44 (1:100, 550,538, BD Biosciences, Franklin Lakes, NJ). After several washes in TBST, slides were incubated in Histofine reagents, which contained an anti‐rat immune‐peroxidase polymer (414311F, Histofine, Nichirei Biosciences, Japan), and then counterstained with Mayer's hematoxylin (TA‐125‐MH, Richard‐Allan Scientific LLC, Kalamazoo, MI) for 30 s. Images were obtained with optical microscopy (Olympus BX51, Hamburg, Germany). The proportion of CD44‐positive glomeruli was calculated by examination of at least 50 glomeruli per cortical section for each mouse, by at least two experimenters.

### 2.5 Immunofluorescence assay and confocal microscopy

Three micrometer thick sections of AFA‐fixed and paraffin‐embedded kidneys were deparaffinized and incubated for 20 min in the target retrieval solution pH 9 (S2367, Dako, Agilent Technologies, Santa Clara, CA) at 100°C in a pressure cooker and blocked in TBS‐0.1% Tween containing 10% bovine serum albumin. Sections were incubated overnight in a humidified chamber at 4°C with anti‐WT1 (1:100, ab89901, Abcam, Cambridge, UK) and anti‐nephrin antibodies (1:100, GP‐N2, Progen, Heidelberg, Germany). The following day, after washings in TBST, sections were incubated for 1 h at room temperature with Alexa Fluor 594‐conjugated donkey anti‐rabbit IgG secondary antibody and Alexa Fluor 488‐conjugated goat anti‐guinea pig IgG secondary antibody, each diluted at 1:750 in TBST. Nuclei were then counterstained with DAPI (1:4000, 62,248, Thermo Fisher Scientific, Waltham, Massachusetts) for 3 min. Images were obtained with an Olympus IX83 with an Andor Revolution DSD2 spinning disk unit. Quantification of the podocytes number per glomerulus was determined by counting the number of WT1‐positive cells in at least 15 glomeruli per mouse. The ratio of the nephrin‐labeled area to the total area (expressed as a percentage) was determined for a minimum of 15 glomeruli per mouse. All images were analyzed and processed using ImageJ software (version 1.53c).

### 2.6 Transmission electron microscopy

Kidneys were fixed in 2.5% glutaraldehyde in 0.1 mol/L cacodylate buffer (pH 7.4) at 4°C for 24 h and then post‐fixed in 1% OsO4 for 1 h, dehydrated using graded alcohol series, and embedded in epoxy resin. Semi‐thin sections (0.5 mm) were stained by toluidine blue, and 60‐nm ultrathin sections were contrasted with uranyless (Delta Microscopies, Mauressac, France) and then lead citrate. Images were obtained with a JEOL 1010 electron microscope (JEOL, Ltd., Tokyo, Japan) with a MegaView III camera (Olympus Soft Imaging Systems GmbH, Munster, Germany). The mean GBM thickness (nm) was measured using a method adapted from (Marquez et al., 2003). For each mouse, at least 2 glomeruli and 10 capillary loops per glomerulus were analyzed. GBM thickness was measured on 10 points per capillary loop by an examiner who was blinded to the experimental conditions. GBM thickness measurements were then averaged for each mouse.

### 2.7 Isolation of glomeruli

Glomeruli were isolated by two‐step sieving of renal cortices. Kidneys were decapsulated, cut into small pieces, and digested for 3 min at 37°C in collagenase I (2 mg/ml, 17,100–017, Gibco, Thermo Fisher Scientific, Waltham, MA, USA) in RPMI 1640 (Gibco, Thermo Fisher Scientific, Waltham, MA, USA). The collagenase I was then inactivated by the addition of RPMI 1640 containing 10% of Fetal Calf Serum (FCS, BioSera, Nuaille, France). The digested tissue was then passed through a 70‐μm cell strainer (BD falcon) on a 50‐ml tube. The filter was flushed with PBS + 0.5% BSA and then discarded. Next, the 50‐ml tube containing tubules and glomeruli was shaken several times and passed through a 40‐μm cell strainer (BD falcon). Glomeruli adherent to the 40‐*μ*m cell strainer were taken from the cell strainer with PBS + 0.5% BSA injected under pressure and then washed in PBS. Isolated glomeruli were then frozen at −80°C.

### 2.8 Total RNA extraction, reverse transcription, and quantitative RT‐PCR

Dry pellets of glomeruli were then lysed and homogenized in TRIzol reagent (MRC, Cincinnati, OH, USA). Phase separation was made using BAN (BN 191, MRC, Cincinnati, OH, USA). RNA was precipitated with isopropanol and washed with 70% ethanol. After air drying, the pellet was resuspended in nuclease‐free water. Following this step, a DNase (EN0521, Thermo Fisher Scientific, Waltham, MA, USA) treatment was performed for 30 min at 37°C. Reverse transcription (K1642, Thermo Fisher Scientific, Waltham, MA, USA) was then made using 200 ng of RNA per sample. Differential gene expression was quantified by real‐time PCR performed in a 96‐well format using the light cycler 480 SYBR Green I Master (Roche Life Science, Meylan, France), the Bio‐Rad Connect Real‐Time detection system apparatus, and the CFX maestro 2.2 software (Version 5.2.008.0222). All the samples were assayed in duplicate, and the average value of the duplicate was used for quantification. Relative gene expression levels were calculated after normalization with housekeeping genes (*Gusb*, *HPRT*, *and RPL32*). Primers for mouse transcripts of *Laminin α5*, *Laminin β2*, *Laminin γ1*, and *Wilm's Tumor 1* were used. Calculations were made using the Pfäffl method (Pfaffl, 2001). The sequences of the primers are listed in Table 1.

**TABLE 1:** Primers sequence for real‐time PCR

Transcript	Forward primer	Reverse primer
mGusb	CTCTGGTGGCCTTACCTGAT	CAGTTGTTGTCACCTTCACCTC
mHPRT	CCTCCTCAGACCGCTTTTT	AACCTGGTTCATCATCGCTAA
mRPL32	GCTGCCATCTGTTTTACGG	TGACTGGTGCCTGATGAACT
mLaminin α5	GCAGGACGACGACGTCATCT	AAGTCTCGAAGTAACGGTGAGTAGGA
mLaminin β2	CAAGCACAATACTCGTGGACTCAA	TCCGACAGGCGTGAGTATGG
mLaminin γ1	AAGGCTGCCAACCCCATCT	AGACCACCGAGCTGAGGATCA
mWT1	CAGATGAACCTAGGAGCTACCTTAAA	TGCCCTTCTGTCCATTTCA

### 2.9 Immunoblot

Total kidney protein lysates from controls and *NPHS2‐Cre* ^*+/−*^ were prepared with RIPA extraction buffer containing phosphatase inhibitors and protease inhibitors (Santa Cruz). Total protein concentration was measured using the BCA protein assay kit (23,225/23227, Thermo Fisher Scientific). Fifty microgram of proteins was loaded onto polyacrylamide electrophoresis gels for separation and transferred onto nitrocellulose membranes. The membranes were blocked with TBS 1X containing 0.1% of Tween (TBST) and 5% of non‐fat milk and then probed with the following antibodies: goat anti‐Nephrin (1:1000, AF3159, R&D) and rabbit anti‐GAPDH (1:20,000, G9545, Sigma). After three 10‐min washing in TBST, membranes were then incubated with horseradish peroxidase‐conjugated secondary antibodies [donkey anti‐rabbit 1/5000 (NA9340, Amersham); donkey anti‐Goat 1/5000 (ab6885, Abcam)]. The resulting bands were visualized by enhanced chemiluminescence (Clarity Western ECL substrate; Bio‐Rad, 170–5061), using a ChemiDoc MP imaging system (12,003,154, Bio‐Rad). Densitometric analysis was used for quantification with the ImageJ software.

### 2.10 Statistical analysis

The statistical test used for the analysis is indicated in the Figure legends. A difference between groups was considered significant when *p* < 0.05. For quantitative variables, we tested the normality of the distribution using the Shapiro–Wilk test. In the case of a Gaussian distribution, a parametric test was used. In the case of heteroskedasticity, Welch's test was performed. Otherwise, an unpaired t‐test was performed. In absence of a normal distribution, a Mann–Whitney test was performed. Comparison of continuous data between two groups was performed with a two‐way ANOVA with Sidak post‐test. The statistical analysis was performed using GraphPad PRISM software v9 (GraphPad Software, La Jolla, CA).

In the figures, the data are represented as individual data, with the larger bar corresponding to the mean and the smaller ones to the standard deviation when the parameter followed a Gaussian distribution, or the median with an interquartile range otherwise.

## 3 RESULTS

### 3.1 Heterozygous C57BL/6J *NPHS2‐Cre* mice do not display a specific renal phenotype at baseline compared with control mice

**FIGURE 1:**
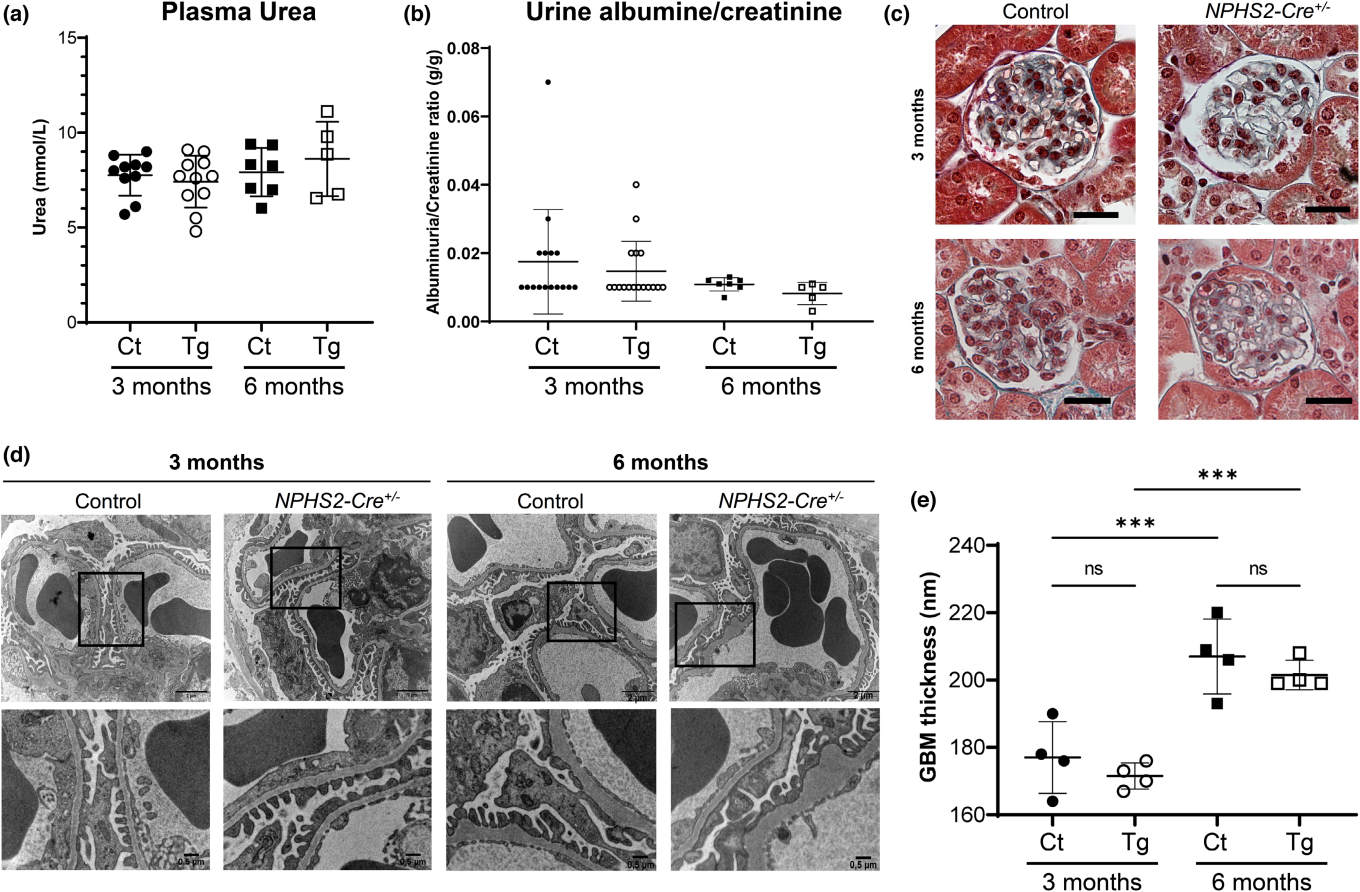
Functional, histopathological, and ultrastructural assessment of control (Ct) and heterozygous *NPHS2‐Cre* ^*+/−*^ (Tg) mice at 3 and 6 months of age. (a) Urea plasma concentration in control and *NPHS2‐Cre* ^*+/−*^ mice (3‐month‐old: *n* = 10 and 11; 6‐month‐old: *n* = 7 and 5, respectively). No statistical difference between the two groups was observed (unpaired *t*‐test). (b) Urinary albumin‐to‐creatinine ratio of control and heterozygous *NPHS2‐Cre* ^*+/−*^ mice at 3 and 6 months of age (One‐way ANOVA, 3‐month‐old: *n* = 10 and 11; 6‐month‐old: *n* = 7 and 5, respectively). No significant albuminuria was observed at baseline. (c) Representative images of Masson trichrome stain of control and *NPSH2‐Cre* ^*+/−*^ glomeruli. No evidence of focal segmental glomerulosclerosis was found in the two groups. Scale bar: 25 μm. (d) Ultrastructural analysis of glomerular basement membrane (GBM) by transmission electron microscopy (TEM). Representative electron micrographs of glomerular sections from control and *NPHS2‐Cre* ^*+/−*^ mice. (e) Quantification of mean GBM thickness (nm) of control and *NPHS2‐Cre* ^*+/−*^ mice (*n* = 4 mice/group). Using a Mann–Whitney test, no difference between control and transgenic mice could be detected but a significant thickening of the GBM between 3 and 6 months of age was observed. ***p < 0.001.

We first verified if the *NPHS2‐Cre* ^*+/−*^ mice commonly used in our laboratory for conditional knock‐out in podocytes presented a renal phenotype at 3 and 6 months of age. Plasma urea concentration and urinary albumin‐to‐creatinine were similar between control littermates and *NPHS2‐Cre* ^*+/−*^ mice at baseline (Figure 1a,b). Similarly, no focal segmental glomerulosclerosis lesion was observed using Masson trichrome stain (Figure 1c).

Previous reports indicated that *NPHS2‐Cre* ^*+/−*^ mice at 24 weeks of age displayed a thickening of their glomerular basement membrane (GBM), detected by transmission electron microscopy (TEM), despite having no obvious renal phenotype or microscopic damage on conventional staining (Balkawade et al., 2019). Using the same technique, we did not observe any thickening of the GBM, in either 3‐ or 6‐month‐old *NPHS2‐Cre* ^*+/−*^ mice, although we did observe a significant thickening of the GBM between the two age groups (Figure 1d,e). Neither foot process effacement nor expansion of GBM was seen.

### 3.2 The mRNA expression of laminin *α*5*β*2*γ*1 subunits is similar between heterozygous C57BL/6J *NPHS2*‐*Cre* mice and their controls

**FIGURE 2:**
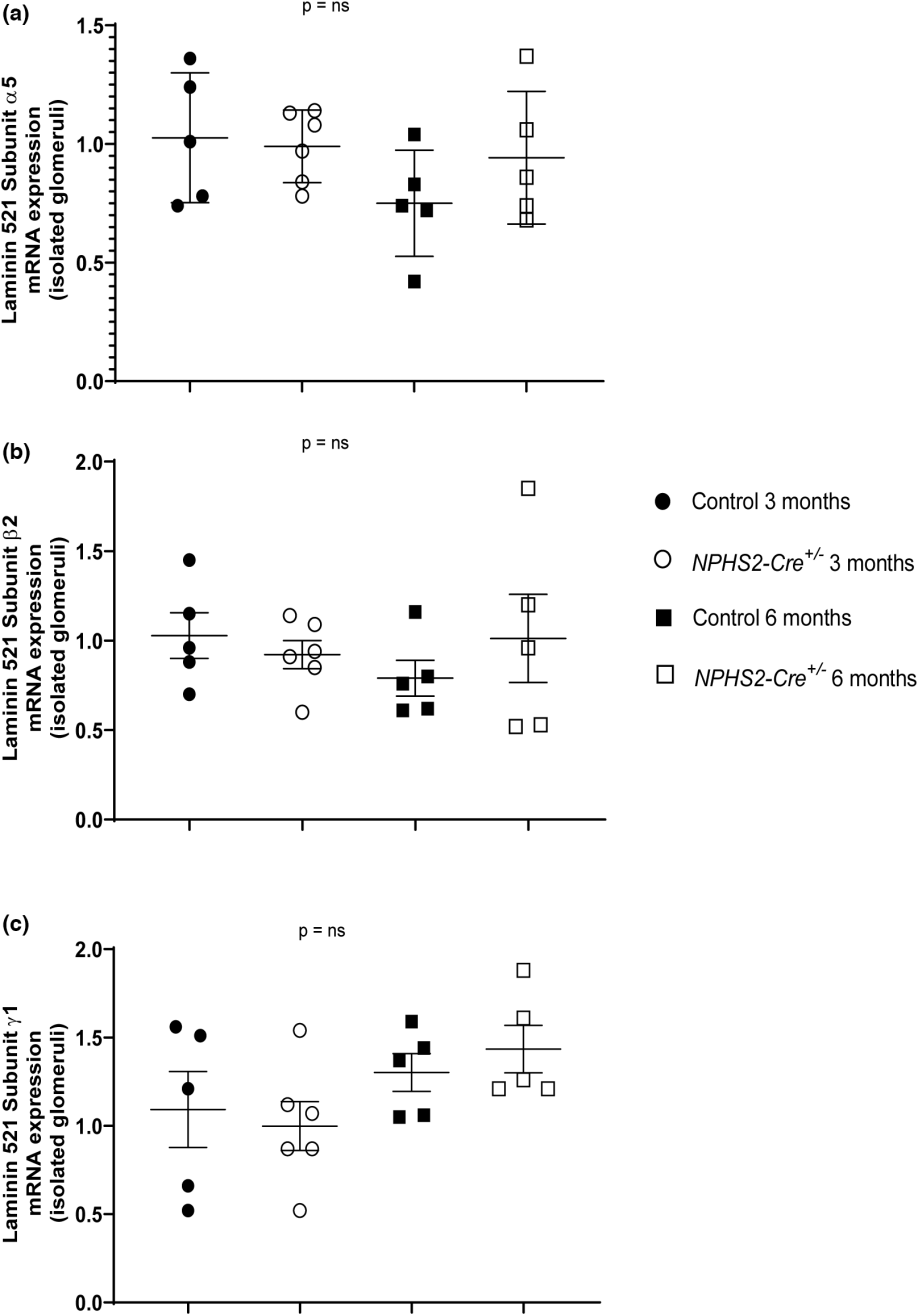
Level of mRNA expression of the individual subunits of laminin 521 is similar between *NPHS2‐Cre* ^*+/−*^ and control mice at baseline, at either 3 or 6 months of age. (a–c) Expression of the three subunits (α5, β2, and γ1) of Laminin 521 in glomeruli of control and *NPHS2‐Cre* ^*+/−*^ mice (3‐month‐old: *n* = 5 and 7; 6‐month‐old: *n* = 5 and 5, respectively). No difference was observed using a Mann–Whitney test. NS, non‐significant.

Previous publication by Balkawade et al. has found that part of the Cre recombinase podotoxicity could be due to altered expression of subunits of laminin *α5β2γ1*, one of the most important components of the GBM. We determined mRNA expression of the three subunits α5, β2, and γ1 that composed mature isoform of the laminin protein in glomeruli isolated from 3‐ and 6‐month‐old control and *NPHS2‐Cre* ^*+/−*^ mice. None of the three subunits were down or upregulated in glomeruli of *NPHS2‐Cre* mice (Figure 2a–c).

### 3.3 Podocyte number and differentiation are not affected by Cre expression in podocytes

**FIGURE 3:**
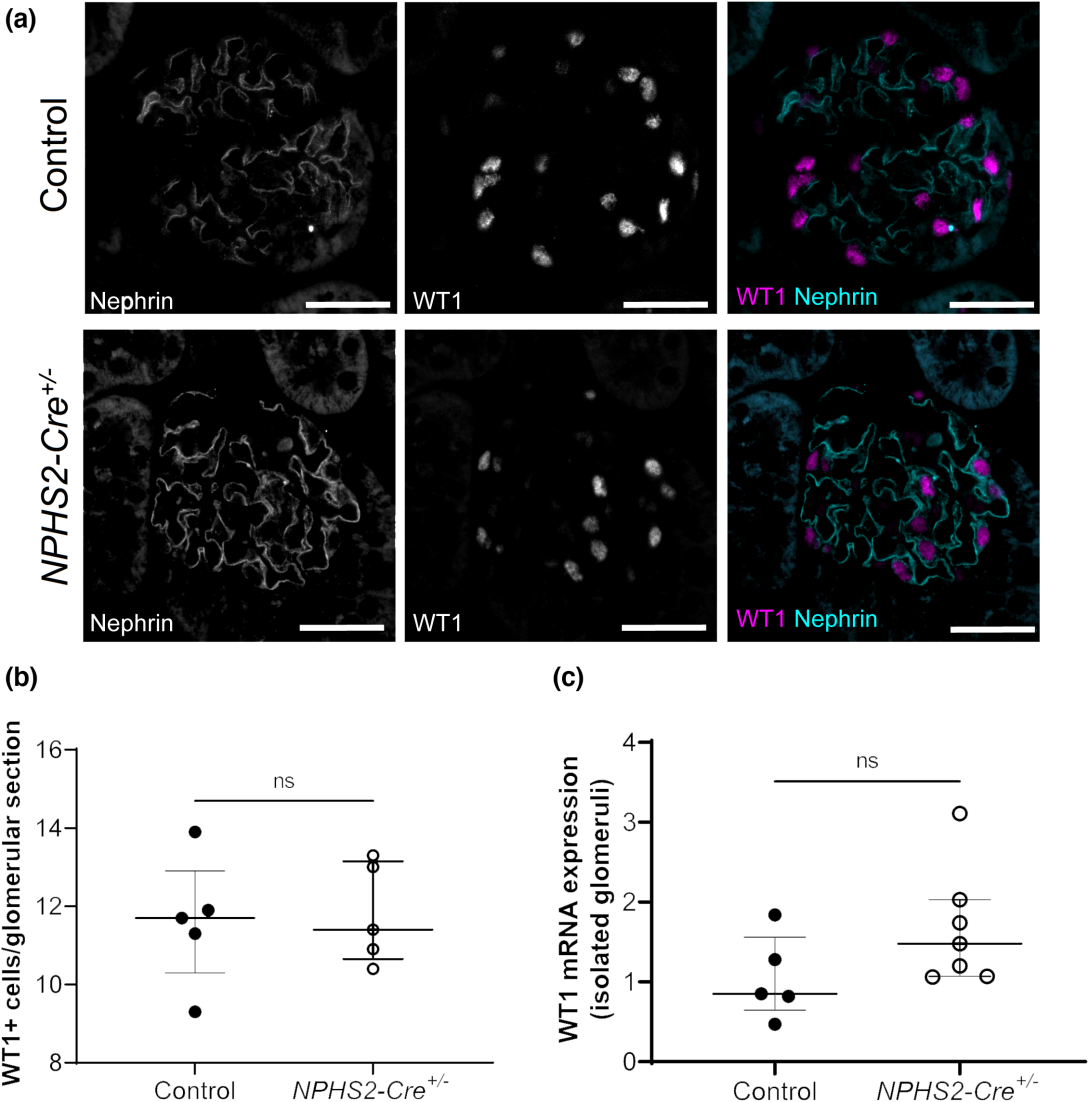
Heterozygous expression of podocyte Cre recombinase does not impact podocyte number or nephrin distribution. (a) Representative images of immunofluorescent staining for Wilms Tumor 1 (WT1) and Nephrin of paraffin‐embedded tissue in control and *NPHS2‐Cre* mice at 8–16 weeks of age. Scale bar (white): 25 μm. (b) Comparison of the average number of WT1 positive cells per glomerulus between control (*n* = 5) and *NPHS2‐Cre* mice (*n* = 5). At least 15 glomeruli per mouse were analyzed. No statistical difference was observed between the two groups using a Mann–Whitney test. C: mRNA expression of WT1 in isolated glomeruli of control (*n* = 5) and *NPSH2‐Cre* (*n* = 7) mice at 10 weeks of age. No statistical difference was observed using a Mann–Whitney test.

Although there was no structural or functional difference between the two groups of mice, we wondered whether an alteration in podocyte number or an alteration of nephrin protein distribution could be seen. Thus, we performed immunofluorescence costaining for Wilm's tumor 1 (WT1) and nephrin in control and *NPHS2‐Cre* ^*+/−*^ mice of 8–16 weeks of age (Figure 3a). No qualitative alteration in nephrin distribution was observed. The number of WT1‐positive glomerular cells was similar between the groups (Figure 3b). This result was confirmed by quantification of the mRNA level of WT1 in isolated control and *NPHS2‐Cre* ^*+/−*^ glomeruli (Figure 3c).

### 3.4 The severity of anti‐GBM‐GN is not impacted by podocyte‐specific Cre expression

**FIGURE 4:**
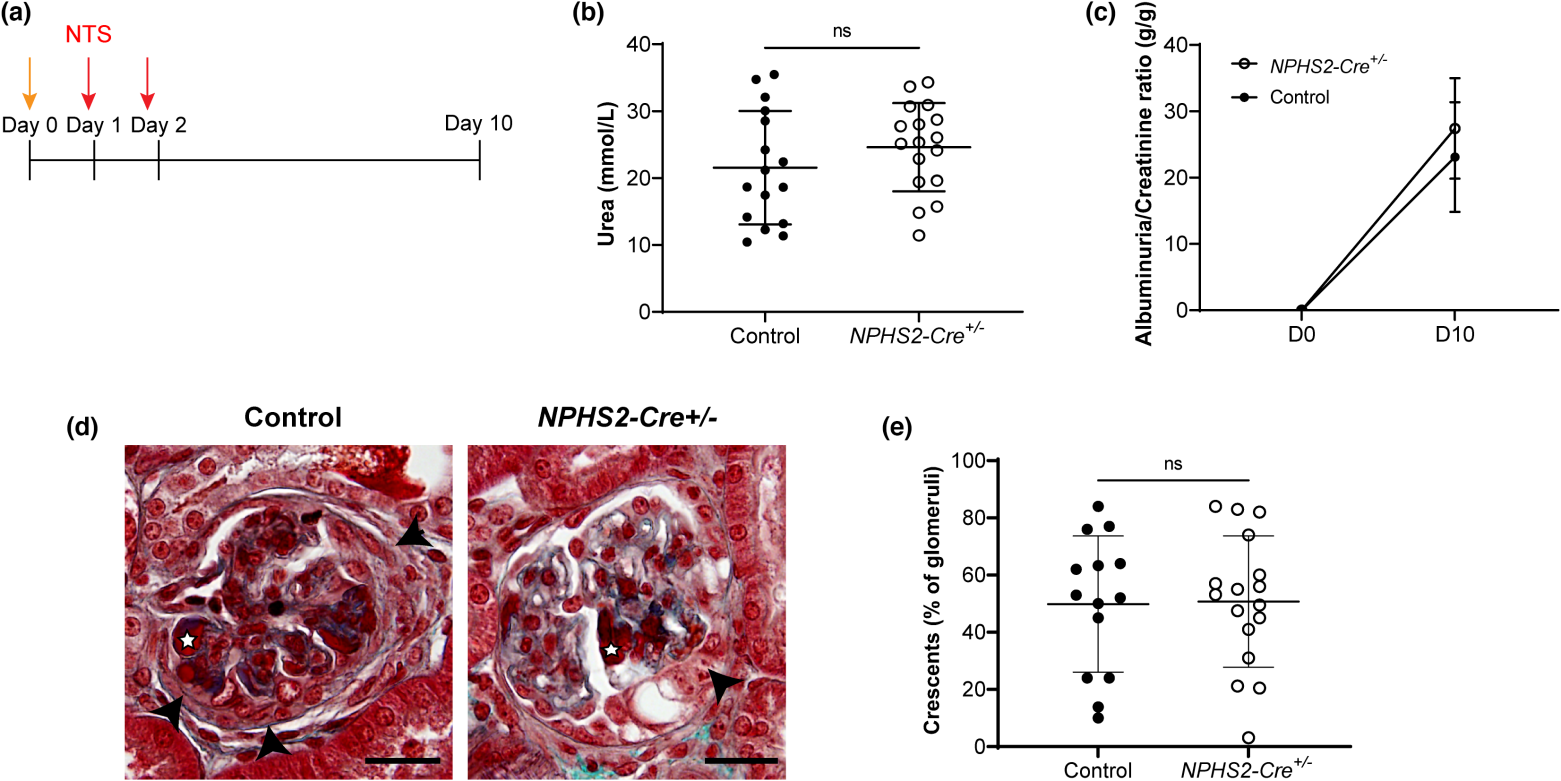
Podocyte‐specific expression of Cre recombinase did not aggravate the renal phenotype after administration of the nephrotoxic serum (NTS). (a) Schematic representation of anti‐GBM‐GN model induced in mice at 12 weeks of age. The range arrow represents the induction dose at Day 0 (50 μl of NTS with 50 μl of physiological serum). The red arrows represent the injection of the full dose of NTS (9 μl/g) on Day 1 and Day 2. (b) Both groups of mice displayed the same degree of plasma urea elevation (unpaired *t*‐test; see Figure 1a for the normal range of plasma urea). (c) While mice of both groups display no basal albuminuria, the urinary albumin/creatinine ratio increased 10 days after NTS injection, but similarly in the two groups (H – two‐way ANOVA with mixed‐effects analysis with a Sidak post‐test). (d) Representative images of Masson trichrome staining showing crescents (black arrowhead) with endocapillary fibrin deposit (white star) in control and *NPHS2‐Cre* ^*+/−*^ mice. Scale bar: 25 μm. (e) Quantification of the percentage of crescentic glomeruli per mouse. At least 50 glomeruli per kidney were analyzed (unpaired *t*‐test). For all the panels, *n* = 14 control and 17 *NPHS2‐Cre* ^*+/−*^ mice (males and females).

Despite the absence of a basal renal phenotype in 2‐ to 4‐month‐old *NPHS2‐Cre* ^*+/−*^ mice, we decided to assess whether these mice were more sensitive to an immune‐mediated severe glomerular insult. For this purpose, we used the anti‐GBM‐GN model, which mimics crescentic glomerulonephritis (Figure 4a). As expected, an acute kidney and glomerular injury was observed, as evidenced by an increase in the plasma concentration of urea as well as in the albuminuria/creatinuria ratio at Day 10. However, there was no significant statistical difference between the two groups for these parameters (Figures 4b,c). One of the hallmarks of anti‐GBM‐GN is the development of crescentic glomeruli, defined by the presence of at least one layer of proliferative cells in the urinary space. *NPHS2‐Cre* ^*+/−*^ mice had the same percentage of crescentic glomeruli as control mice on Day 10 (Figures 4d,e).

### 3.5 Activation of parietal epithelial cells and expression of key podocyte proteins are not modified in NPHS2‐Cre mice in anti‐GBM‐GN model

**FIGURE 5:**
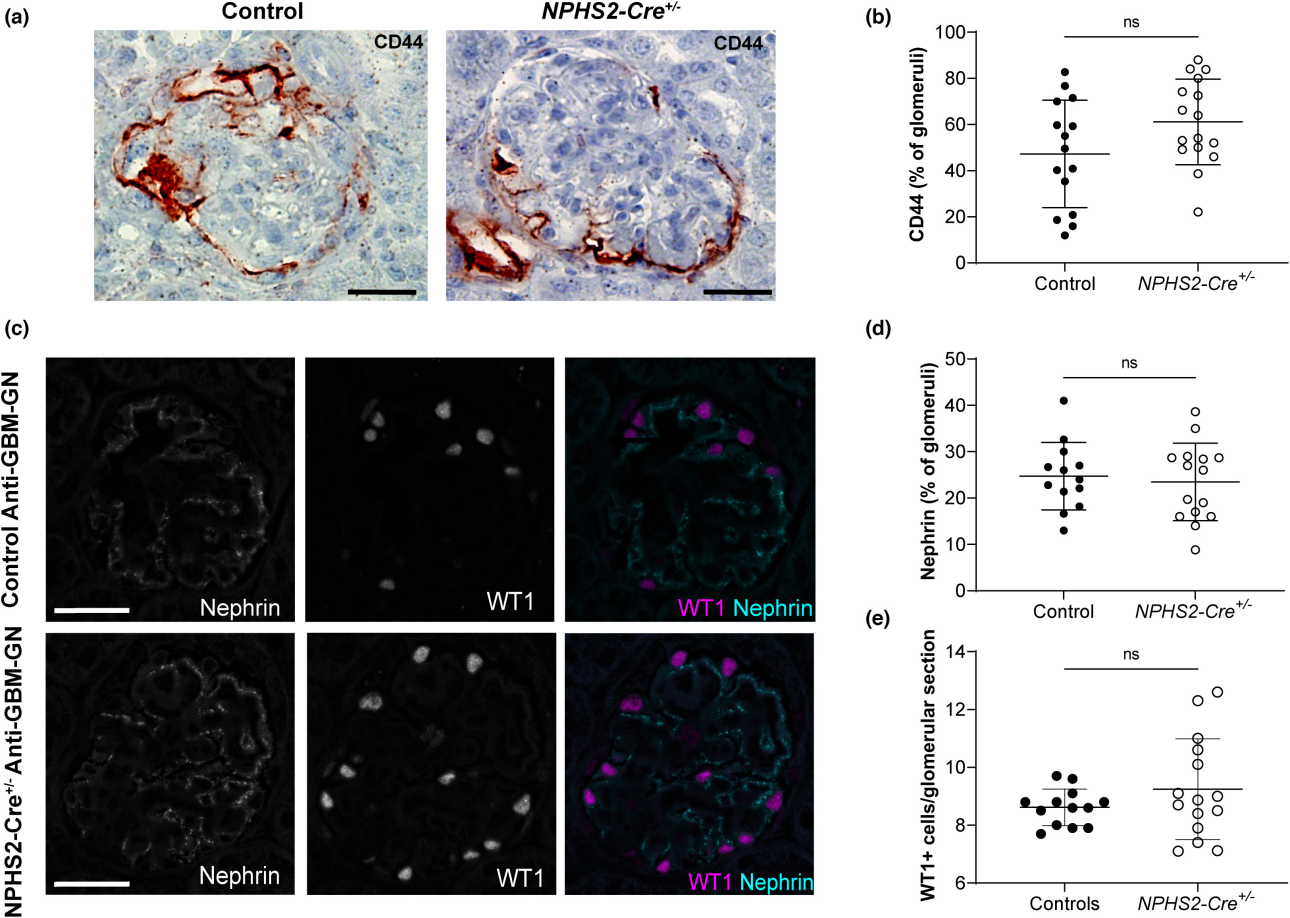
Podocyte‐specific expression of the Cre recombinase did not increase the activation of parietal epithelial cells or the alteration of key podocyte proteins during anti‐GBM‐GN. (a) Representative images of CD44 immunochemistry counterstained with hematoxylin in control and *NPHS2‐Cre* ^*+/−*^ mice (scale bar: 25 μm). (b) No difference in terms of percentage of CD44‐positive glomeruli was observed. (Unpaired *t*‐test ‐ control: *n* = 15—*NPHS2‐Cre* ^*+/−*^: *n* = 17). (c) Representative images of immunofluorescence costaining for Nephrin (cyan) and WT1 (magenta) in control and *NPHS2‐Cre* ^*+/−*^ mice affected by anti‐GBM‐GN. In both groups, nephrin redistribution was noted. (d) No difference in nephrin expression within the glomeruli was observed between the two groups (Unpaired *t*‐test—control: *n* = 13—*NPHS2‐Cre* ^*+/−*^: *n* = 15). (e) The number of WT1‐positive podocytes was not different between the two groups (Welch's *t*‐test—control: *n* = 13—*NPHS2‐Cre* ^*+/−*^: *n* = 15).

To verify whether subclinical changes occur in *NPHS2‐Cre* ^*+/−*^ mice in pathological conditions, we quantified the expression of three key proteins involved in the anti‐GBM‐GN model. One of the major cell types involved in crescent formation is the parietal epithelial cells. When activated, it expresses the membrane marker CD44. By immunohistochemistry directed against CD44, we did not find any difference in the number of CD44‐positive glomeruli between the two groups (Figure 5a,b). We also evaluated the expression of WT1 and nephrin by immunofluorescence costaining (Figure 5c). In both groups, a punctiform redistribution of nephrin was observed and the nephrin‐positive surface was similar between the two groups (Figure 5d). The lack of impact of podocyte Cre recombinase expression on nephrin abundance was confirmed by immunoblot (Figure S1). Similarly, there was no difference in the number of WT‐1 positive cells per glomeruli between the groups (Figure 5e).

## 4 DISCUSSION

In this study, we first show that the expression of Cre in the podocytes does not modify the renal phenotype of young heterozygous *NPHS2‐Cre* ^*+/−*^ adult mice at baseline. In addition, imposing a glomerular insult on these mice does not unmask a more severe phenotype.

Although the *NPHS2‐Cre* transgenic mouse line has been used for conditional knock‐out in podocytes for over 15 years (Moeller et al., 2003), the first report of its toxicity in these cells was published only in 2019 (Balkawade et al., 2019). A global thickening of the GBM was found in heterozygous and homozygous *NPHS2‐Cre* without overt clinical phenotype in 6‐month‐old animals. The podotoxicity of the Cre recombinase was then confirmed in a mouse transgenic model of doxycycline‐inducible podocyte‐specific Cre expression, in which the same *NPHS2* promoter was used to direct the expression of the tetracycline‐controlled transcriptional activator (rtTA) in the podocytes (Frahsek et al., 2019). When doxycycline was administered 3 or 4 days after birth (but not later), a progressive loss of podocytes and the development of focal segmental glomerulosclerosis were observed between 8 days and 13 weeks of age (Frahsek et al., 2019). The histological lesions were more severe than in the first report and associated with proteinuria and renal failure. Therefore, previous studies using the *NPHS2‐Cre* strain could have been flawed in the absence of an appropriate control group (e.g., expressing only the Cre recombinase of interest without the floxed alleles of the gene of interest).

Given that we are commonly using the *NPHS2‐Cre* lines to assess the role of proteins of interest in the context of glomerular diseases in 2‐to 4‐month‐old animals, we decided to characterize the phenotype of heterozygous *NPHS2‐Cre* mice at baseline and in pathological conditions in young adult mice. Our study shows that 2‐ to 4‐month‐old *NPHS2‐Cre* ^*+/−*^ mice do not present a clinical phenotype and have no alteration of the glomerular ultrastructure in both conditions. Balkawade and collaborators observed the modified glomerular phenotype in 6‐month‐old animals. However, we did not observe any difference between the transgenic animals and their control littermates at this age, at baseline. The discrepancy between our report and the previous ones could be due to the genetic background. Indeed, we used *NPHS2‐Cre* ^*+/−*^ mice on a C57BL/6J background and not on a 129S6/SvEvTac, as in the other two publications. The difference between these two genetic backgrounds in both physiological and pathological conditions is now well established. In the context of glomerular pathophysiology, Mesnard and collaborators have shown that the administration of NTS induced a more severe glomerular disease in 129Sv mice than in C57BL/6J mice. In particular, glomerular thrombotic microangiopathy (TMA) was significantly more frequently observed in 129Sv mice. This is probably caused by a lower basal glomerular endothelial *Vegfr2* expression level in 129Sv mice than in C57BL/6J mice, as blocking VEGFR2 signaling aggravates the consequences of NTS administration only in C57BL/6J mice (Mesnard et al., 2014).

Cre recombinase toxicity is commonly attributed to the presence of cryptic LoxP sequences in the genome (Thyagarajan et al., 2000) but also to its endonuclease activity (Schmidt et al., 2000). The fact that the physiology of podocytes is modified in two independent transgenic lines strongly suggests that the expression of the Cre recombinase is solely responsible for these changes and not the site of insertion of the transgene. The more severe phenotype observed in the doxycycline‐inducible transgenic animals could be due to a higher level of expression of the Cre recombinase. Indeed, the FSGS lesions only appear in these mice when the *LC1* transgene, which carries the Cre sequence, is present at the homozygous stage (Frahsek et al., 2019). In addition, increasing the dose of doxycycline results in a more severe phenotype. This dose‐dependent toxicity of Cre has also been well documented in vitro *(*Baba et al., 2005; Loonstra et al., 2001). A comparison of the level of expression between the two transgenic lines (inducible and constitutive) would be required to confirm this hypothesis.

We found 137 studies indexed in PubMed that used the *NPHS2‐Cre* transgenic line to characterize the role of genes of interest in the podocytes. In 14.6% of these studies, no detectable effect was observed on the podocyte and/or glomerulus physiology. In 59.1% of them, a deleterious phenotype was observed, either spontaneously (38.7%) or after induction of pathological conditions (20.4%; adriamycin‐induced FSGS, diabetes, anti‐GBM‐GN…). Finally, the remaining studies (26.3%) described a protective effect of the inactivation of the gene of interest in the aforementioned pathological conditions.

Taken together, this last group of studies and ours strongly suggest that the *NPHS2‐Cre* transgene can be safely used in glomerular disease models for podocyte‐specific gene targeting in young C57BL/6J adult mice.

## AUTHOR CONTRIBUTIONS

CM and JH conceived and designed research, interpreted results of experiments, prepared figures, drafted manuscript, edited and revised manuscript, and approved the final version of the manuscript. CM, EL, CN, MCV, and JH performed experiments. CM, TM, PF, EL, and CN analyzed the data.

## FUNDING INFORMATION

This work was supported by the Institut National de la Santé et de la Recherche Médicale (Inserm). Juliette Hadchouel received a grant from the Académie de Médecine, sponsored by Nestlé Waters France. Cyril Mousseaux received a PhD fellowship from the Fondation pour la Recherche Médicale (Grant # FDM201906008792).

## Supporting information


**Appendix S1** Supplementary Information

## References

[phy215443-bib-0001] Almaani, S. , Meara, A. , & Rovin, B. H. (2017). Update on lupus nephritis. Clinical Journal of the American Society of Nephrology, 12, 825–835. 10.2215/CJN.05780616 27821390 PMC5477208

[phy215443-bib-0002] Baba, Y. , Nakano, M. , Yamada, Y. , Saito, I. , & Kanegae, Y. (2005). Practical range of effective dose for cre recombinase‐expressing recombinant adenovirus without cell toxicity in mammalian cells. Microbiology and Immunology, 49, 559–570. 10.1111/j.1348-0421.2005.tb03753.x 15965304

[phy215443-bib-0003] Balkawade, R. S. , Chen, C. , Crowley, M. R. , Crossman, D. K. , Clapp, W. L. , Verlander, J. W. , & Marshall, C. B. (2019). Podocyte‐specific expression of Cre recombinase promotes glomerular basement membrane thickening. American Journal of Physiology Renal Physiology, 316, F1026–F1040. 10.1152/ajprenal.00359.2018 30810063 PMC7002867

[phy215443-bib-0004] Dai, Y. , Gu, L. , Yuan, W. , Yu, Q. , Ni, Z. , Ross, M. J. , Kaufman, L. , Xiong, H. , Salant, D. J. , He, J. C. , & Chuang, P. Y. (2013). Podocyte‐specific deletion of signal transducer and activator of transcription 3 attenuates nephrotoxic serum‐induced glomerulonephritis. Kidney International, 84, 950–961. 10.1038/ki.2013.197 23842188 PMC3797218

[phy215443-bib-0005] Frahsek, M. , Schulte, K. , Chia‐Gil, A. , Djudjaj, S. , Schueler, H. , Leuchtle, K. , Smeets, B. , Dijkman, H. , Floege, J. , & Moeller, M. J. (2019). Cre recombinase toxicity in podocytes: a novel genetic model for FSGS in adolescent mice. American Journal of Physiology Renal Physiology, 317, F1375–F1382. 10.1152/ajprenal.00573.2018 31588799

[phy215443-bib-0006] Jeannotte, L. , Aubin, J. , Bourque, S. , Lemieux, M. , Montaron, S. , & Provencher, S.‐P. A. (2011). Unsuspected effects of a lung‐specific Cre deleter mouse line. Genesis, 49, 152–159. 10.1002/dvg.20720 21309069

[phy215443-bib-0007] Jennette, J. C. , & Nachman, P. H. (2017). ANCA glomerulonephritis and vasculitis. Clinical Journal of the American Society of Nephrology, 12, 1680–1691. 10.2215/CJN.02500317 28842398 PMC5628710

[phy215443-bib-0008] Lee, J.‐Y. , Ristow, M. , Lin, X. , White, M. F. , Magnuson, M. A. , & Hennighausen, L. (2006). RIP‐Cre revisited, evidence for impairments of pancreatic beta‐cell function. The Journal of Biological Chemistry, 281, 2649–2653. 10.1074/jbc.M512373200 16326700

[phy215443-bib-0009] Loonstra, A. , Vooijs, M. , Beverloo, H. B. , Allak, B. A. , van Drunen, E. , Kanaar, R. , Berns, A. , & Jonkers, J. (2001). Growth inhibition and DNA damage induced by Cre recombinase in mammalian cells. Proceedings of the National Academy of Sciences of the United States of America, 98, 9209–9214. 10.1073/pnas.161269798 11481484 PMC55399

[phy215443-bib-0010] Marquez, B. , Zouvani, I. , Karagrigoriou, A. , Anastasiades, E. , Pierides, A. , & Kyriacou, K. (2003). A simplified method for measuring the thickness of glomerular basement membranes. Ultrastructural Pathology, 27, 409–416. 14660279

[phy215443-bib-0011] McAdoo, S. P. , & Pusey, C. D. (2017). Anti‐glomerular basement membrane disease. Clinical Journal of the American Society of Nephrology, 12, 1162–1172. 10.2215/CJN.01380217 28515156 PMC5498345

[phy215443-bib-0012] Mesnard, L. , Cathelin, D. , Vandermeersch, S. , Rafat, C. , Luque, Y. , Sohier, J. , Nochy, D. , Garcon, L. , Callard, P. , Jouanneau, C. , Verpont, M.‐C. , Tharaux, P.‐L. , Hertig, A. , & Rondeau, E. (2014). Genetic background‐dependent thrombotic microangiopathy is related to vascular endothelial growth factor receptor 2 signaling during anti‐glomerular basement membrane glomerulonephritis in mice. The American Journal of Pathology, 184, 2438–2449. 10.1016/j.ajpath.2014.05.020 25005449

[phy215443-bib-0013] Mesnard, L. , Keller, A. C. , Michel, M.‐L. , Vandermeersch, S. , Rafat, C. , Letavernier, E. , Tillet, Y. , Rondeau, E. , & Leite‐de‐Moraes, M. C. (2009). Invariant natural killer T cells and TGF‐beta attenuate anti‐GBM glomerulonephritis. Journal of the American Society of Nephrology, 20, 1282–1292. 10.1681/ASN.2008040433 19470687 PMC2689902

[phy215443-bib-0014] Moeller, M. J. , Sanden, S. K. , Soofi, A. , Wiggins, R. C. , & Holzman, L. B. (2003). Podocyte‐specific expression of cre recombinase in transgenic mice. Genesis, 35, 39–42. 10.1002/gene.10164 12481297

[phy215443-bib-0015] Pace, J. A. , Bronstein, R. , Guo, Y. , Yang, Y. , Estrada, C. C. , Gujarati, N. , Salant, D. J. , Haley, J. , Bialkowska, A. B. , Yang, V. W. , He, J. C. , & Mallipattu, S. K. (2021). Podocyte‐specific KLF4 is required to maintain parietal epithelial cell quiescence in the kidney. Science Advances, 7, eabg6600. 10.1126/sciadv.abg6600 34516901 PMC8442927

[phy215443-bib-0016] Pfaffl MW . A new mathematical model for relative quantification in real‐time RT–PCR. Nucleic Acids Research 29: e45, 2001, 45e, 445. 11328886 10.1093/nar/29.9.e45 PMC55695

[phy215443-bib-0017] Salant, D. J. , & Cybulsky, A. V. (1988). Experimental glomerulonephritis. Methods in Enzymology, 162, 421–461. 10.1016/0076-6879(88)62096-9 3265756

[phy215443-bib-0018] Sauer, B. , & Henderson, N. (1988). Site‐specific DNA recombination in mammalian cells by the Cre recombinase of bacteriophage P1. Proceedings of the National Academy of Sciences of the United States of America, 85, 5166–5170. 10.1073/pnas.85.14.5166 2839833 PMC281709

[phy215443-bib-0019] Schmidt, E. E. , Taylor, D. S. , Prigge, J. R. , Barnett, S. , & Capecchi, M. R. (2000). Illegitimate Cre‐dependent chromosome rearrangements in transgenic mouse spermatids. Proceedings of the National Academy of Sciences of the United States of America, 97, 13702–13707. 10.1073/pnas.240471297 11087830 PMC17639

[phy215443-bib-0020] Sternberg, N. , & Hamilton, D. (1981). Bacteriophage P1 site‐specific recombination. I. Recombination between loxP sites. Journal of Molecular Biology, 150, 467–486. 10.1016/0022-2836(81)90375-2 6276557

[phy215443-bib-0021] Thyagarajan, B. , Guimarães, M. J. , Groth, A. C. , & Calos, M. P. (2000). Mammalian genomes contain active recombinase recognition sites. Gene, 244, 47–54. 10.1016/S0378-1119(00)00008-1 10689186

