# Supplementary material for: Heterozygous expression of Cre recombinase in podocytes has no impact on the anti‐glomerular basement membrane glomerulonephritis model in C57BL/6J mice
Source: Physiol Rep. 2022 Sep 9;10(17):e15443. doi: 10.14814/phy2.15443 (PMC9461343; doi:10.14814/phy2.15443)
Supplement: Supplementary file 1 — Appendix S1 Supplementary Information [file PHY2-10-e15443-s001.docx]

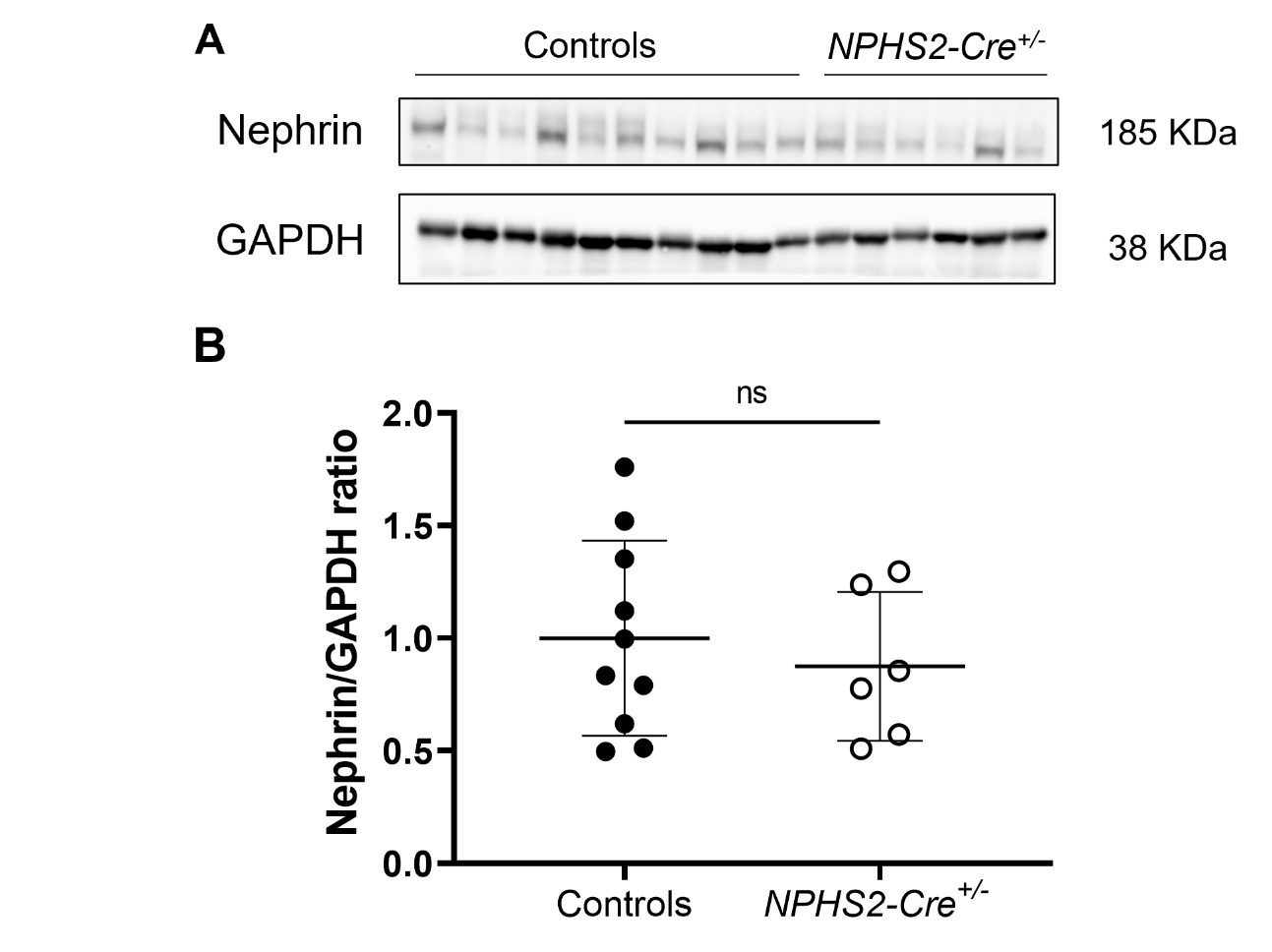


**Supplemental Figure 1. Nephrin abundance was not impacted by podocyte Cre recombinase expression following anti-GBM GN induction.** A. Western blot analysis of the expression of nephrin in control and NPHS2-Cre^+/-^ animals exposed to anti-GBM serum. GAPDH serves as a housekeeping protein. B. Quantification of Nephrin/GAPDH ratio. The data represents the mean +/- standard deviation (Unpaired t-test). n= 10 for control group and n= 6 for NPHS2-Cre^+/-^ group.
